# Effects of Low-Flux and High-Flux Dialysis Membranes on Erythropoietin Responsiveness in Hemodialysis Patients

**DOI:** 10.1155/2022/2984193

**Published:** 2022-06-14

**Authors:** Walid A. R. Abdelhamid, Mohamed M. Soliman, Ayman R. A. El-Hameed

**Affiliations:** ^1^Department of Internal Medicine, Zagazig University, Zagazig, Egypt; ^2^Department of Nephrology, Ibra Hospital, Ibra, Oman

## Abstract

**Background:**

Chronic kidney disease (CKD) is often accompanied by anemia. High-flux membranes contribute to a reasonable removal of uremic toxins which cause anemia in CKD. Inadequate data have described the efficiency of high-flux dialysis in promoting erythropoietin responsiveness in CKD patients in the Middle East. This study was conducted to compare the efficiency of maintaining high-flux hemodialysis versus low-flux dialysis for ≥1 year in promoting erythropoietin responsiveness and to show the factors associated with erythropoietin hyporesponsiveness in Arab chronic hemodialysis patients.

**Methods:**

It was a retrospective cohort study that involved 110 subjects who were categorized into group 1 (50 patients receiving low-flux dialysis) and group 2 (60 patients receiving high-flux dialysis). History taking, examination, and laboratory investigations were conducted for all patients every 3 months from January 2021 to January 2022.

**Results:**

Group 2 had significantly higher weight and body mass index than group 1 but lower cholesterol and intact parathyroid hormone levels than group 1. Erythropoietin resistance index levels did not differ between the two groups upon repeated measures over a 1-year follow-up. Significant risk factors for erythropoietin hyporesponsiveness on multivariate analysis were lower weight (Odds ratio (OR): 0.966; 95% Confidence interval (CI): 0.94–0.992; *p*=0.011), longer hemodialysis vintage (OR: 1.172; 95% CI: 1.036–1.325; *p*=0.012), lower hemoglobin levels (OR: 0.531; 95% CI: 0.362–0.779; *p*=0.001), and higher neutrophil-to-lymphocyte ratio (OR: 2.436; 95% CI: 1.321–4.493; *p*=0.004).

**Conclusion:**

High-flux dialysis was not superior to low-flux dialysis in improving erythropoietin responsiveness.

## 1. Introduction

Chronic renal impairment is often accompanied by anemia, which is linked to a higher risk of memory deficits, hospital admission, acute cardiovascular events, and shorter life expectancy. In addition, severe anemia is a poor prognostic factor for patients with chronic kidney disease (CKD) [1]. In contrast, higher hematocrit levels in hemodialysis patients are not safe and increase the risk of mortality in hemodialysis patients [2].

The principal reason for anemia in CKD patients is the accumulation of uremic toxins, which suppress erythropoietin production by renal interstitial fibroblasts [3]. Other elements that have been recognized as attributing to anemia in CKD patients are erythropoietin resistance, systemic inflammation prompted by CKD and associated medical conditions, iron deficiency, higher levels of hepcidin, folate insufficiency, and cobalamin deficit [4].

Dialyzers are generally categorized into low-flux dialyzers and high-flux dialyzers. Low-flux membranes eliminate small solutes via diffusion, but larger solutes are more poisonous and difficult to get rid of them via diffusion [5]. The innovation of high-flux membranes has contributed to reasonable elimination of uremic poisons, particularly those with a molecular weight of 500 Da or above [6]. In developing countries, both types of dialyzers are widely used especially in the Middle East. Therefore, our study was conducted to determine the efficiency of maintaining high-flux hemodialysis versus low-flux dialysis for ≥1 year in promoting erythropoietin responsiveness in Arab patients with end-stage kidney disease and to identify the factors associated with erythropoietin hyporesponsiveness.

## 2. Materials and Methods

The research was executed following Helsinki Declaration's ethical regulations. Before joining the study, each participant provided a written informed consent form. The study was authorized by the Institutional Review Board of the Ethical Committee of Zagazig University (ZU-IRB #9321).

This retrospective cohort study was conducted from January 2021 to January 2022. Criteria to recruit participants in our study were age 18 years or above, chronic kidney disease, regular hemodialysis for at least 1 year, and receiving erythropoietin therapy for 1 year or longer. We omitted patients with active malignancy, active infection, hemoglobinopathies, severe cardiovascular disease, or advanced cirrhosis. The patients were classified into two groups:

- Group 1: it involved patients who underwent regular hemodialysis with low-flux polysulfone dialyzers. There were 50 participants with a mean age ± standard deviation of 55.4 ± 15.3 years.
- Group 2: it involved patients who underwent regular hemodialysis with high-flux polysulfone dialyzers. There were 60 participants with a mean age ± standard deviation of 51 ± 12 years.

Each study participant underwent a thorough medical history taking and clinical examination including the history of comorbid diseases, average interdialytic weight gain (IDWG), the type of vascular access either the permcath or the arteriovenous fistula, and dialysis vintage. In addition, laboratory investigations were collected predialysis except for urea which was collected before dialysis and after dialysis. Investigations consisted of liver function tests, bone profile, estimated glomerular filtration rate using the CKD-EPI (Chronic Kidney Disease Epidemiology Collaboration) equation, urea reduction ratio, complete blood count, iron profile, lipid profile, serum folate level, serum vitamin B12 level, and erythropoietin resistance index (ERI). The ERI was calculated by dividing the average weekly erythropoietin (EPO) dose by body weight by the average hemoglobin over 3 successive months. Patients were administered a treatment protocol using intravenous epoetin *β*. The target hemoglobin level was 10–12 gm/dL. In addition, iron stores of the patients were replenished using intravenous iron sucrose when transferrin saturation was less than 30%, or ferritin was less than 500 ng/mL.

All hemodialysis sessions of the participants were exclusively performed using synthetic polysulfone dialyzers (Helixone; Fresenius Medical Care AG & Co. KGaA, Bad Homburg, Germany). In low-flux dialysis, surface areas of the dialysis membranes were between 1.0 and 1.8 m^2^ (Fresenius FX5 to FX10, INLINE steam sterile). Surface areas of high-flux dialysis membranes were 1.4 and 1.8 m^2^ (Fresenius Classix FX60 and FX80, INLINE steam sterile). The selection of surface areas of dialyzers was adjusted according to the body surface areas of the patients. Dialysis sessions for all participants of the study were three times per week with the blood flow rate at 250–350 ml/min and dialysate flow rate at 500–600 mL/min in low-flux dialysis and 700–800 mL/min in high-flux dialysis.

### 2.1. Data Analysis

Continuous data were checked for normality by utilizing the Shapiro–Wilk test. The *t*-test was utilized to compare continuous parametric data, which were represented as means and standard deviations. The Mann–Whitney *U* test was utilized to compare nonparametric continuous data, which were represented as medians and interquartile ranges (IQR). Frequency counts and percentages were utilized to represent categorical variables. The average ERI over 1 year was classified into high ERI (ERI ≥18 IU/kg/week/g/dl) and low ERI (<18 IU/kg/week/g/dl). Independent predictors of erythropoietin hyporesponsiveness (high ERI group) were defined using univariate binary logistic regression analysis of the baseline variables at the start of the study. Then, variables with *p* ≤ 0.2 were analyzed using multivariate forward logistic regression analysis. Repeated measures of ERI and hemoglobin were analyzed using the general linear model (GLM) which considers within subject effects. SPSS software for Windows, version 26, was used to perform all statistical analyses.

## 3. Results

**Figure 1:**
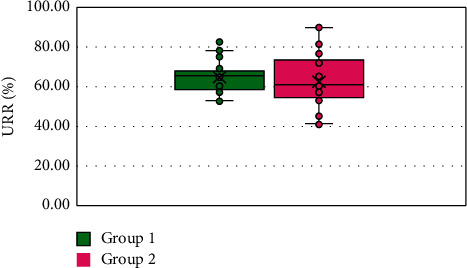
The boxplot graph displays urea reduction ratio (URR) in group 1 and group 2.

**Figure 2:**
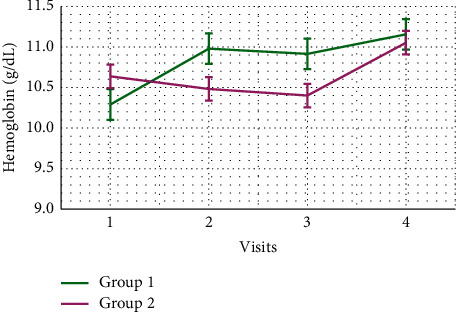
Comparison of hemoglobin levels between group 1 and group 2 over 1 year period. The error bars represent the standard errors of measurements.

**Figure 3:**
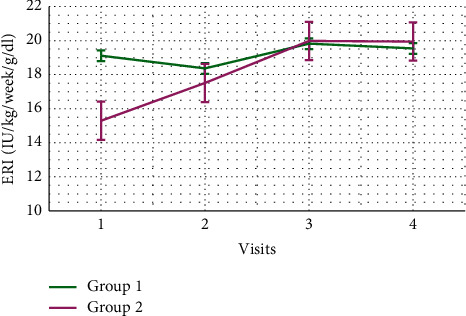
Comparison of erythropoietin resistance index levels between group 1 and group 2 over 1 year period. The error bars represent the standard errors of measurements.

Demographic data are demonstrated in Table 1. The research incorporated 110 participants who were organized into two groups. There were no significant differences regarding age, average systolic blood pressure, average diastolic blood pressure, IDWG, or hemodialysis vintage. Patients in group 2 were primarily males (80%). Furthermore, group 2 had significantly higher weight and body mass index (BMI) than group 1 (Table 1). Moreover, serum cholesterol and intact parathyroid hormone (iPTH) levels were lower in group 2 (Table 2). Urea reduction ratio (URR) levels were not significantly different between the two groups (Figure 1). In addition, significant risk factors for erythropoietin hyporesponsiveness on univariate analysis were lower IDWG, lower serum albumin, lower hemoglobin, higher neutrophil-to-lymphocyte ratio (NLR), lower serum iron, and higher serum ferritin levels. Other marginally significant risk factors included weight, hemodialysis vintage, serum uric acid levels, white blood cell (WBC) counts, neutrophil counts, platelet-to-lymphocyte ratio (PLR), and serum cholesterol levels (Table 3). Then, on multivariate analysis, significant predictors for erythropoietin hyporesponsiveness included lower weight (odds ratio (OR): 0.966; 95% confidence interval (CI): 0.94–0.992; *p*=0.011), longer hemodialysis vintage (OR: 1.172; 95% CI: 1.036–1.325; *p*=0.012), lower hemoglobin (OR: 0.531; 95% CI: 0.362–0.779; *p*=0.001), and higher NLR (OR: 2.436; 95% CI: 1.321–4.493; *p*=0.004) (Table 4). Repeated measures of hemoglobin levels in group 1 and group 2 over 1 year were significantly different using GLM analysis with *p*=0.045 (Figure 2). Finally, repeated measures of ERI levels in group 1 and group 2 over 1 year were not significantly different using GLM analysis with *p*=0.325 (Figure 3).

## 4. Discussion

The purpose of this clinical study was to evaluate the impact of low-flux versus high-flux hemodialysis on erythropoietin resistance in end-stage kidney disease patients receiving regular dialysis therapy for at least one year. We assumed that high-flux dialysis could boost hemoglobin levels and reduce erythropoietin resistance if it was continued regularly for one year or more. Mean hemoglobin was 11.2 g/dL and 11 g/dL in low-flux and high-flux dialysis groups, respectively. Of note, recent proofs demonstrate that there is an evident advantage in adjusting hemoglobin (Hb) levels if they are less than 10 g/dL, but there is also a higher risk when Hb levels are 13 g/dl. Then, the Hb goal seems to be from 10 to 12 g/dl [7], and all participants in our study achieved that target. We detected no discrepancy of ERI levels over 1 year period between the high-flux dialysis and the low-flux dialysis, and these results are consistent with those of Schneider et al. [8].

In addition, high-flux dialysis was associated with higher body weight and BMI. A higher BMI predicts a better outcome in chronic dialysis patients, as it indicates better nutritional status [9]. In addition, we found that high-flux dialysis was associated with lower levels of serum cholesterol. In line with our results, Saini et al. [10] reported the efficiency of hemodialysis in the clearance of cholesterol and triglycerides. In addition, levels of iPTH were lower in group 2 than those in group 1. In line with our results, Li et al. [11] reported similar results. In contrast, Jean et al. [12] observed no marked alterations in serum iPTH levels after switching from conventional hemodialysis to hemodiafiltration, and this may be due to the small sample size of that study (51 patients).

We analyzed the probable predictors for high ERI, and we found that lower weight was a significant risk factor on multivariate analysis. Similar findings were recorded by Hejaili et al. [13]. Generally, lower weight and hypoalbuminemia indicate associated protein-energy malnutrition [14] that induces inflammation and erythropoietin hyporesponsiveness [15]. In addition, longer hemodialysis vintage was another important risk factor. This is consistent with findings in 3591 patients from a retrospective observational study by Rosati et al. [16] and in 1015 patients from a post hoc analysis by Schneider et al. [17]. This can be explained by the fact that longer dialysis is associated with chronic inflammation, accumulation of advanced-glycation end products, endothelial dysfunction, and dysregulated calcium and phosphate homeostasis [18].

Other risk factors of high ERI included lower hemoglobin and high NLR. This is consistent with findings in 299 patients from a recent study by Zhang et al. [19]. This may be supported by that patients with high NLR have higher levels of neutrophils which stick to and infiltrate the endothelial wall and release inflammatory cytokines (especially interleukin-1 and tumor necrosis factor alpha) [20], free oxygen radicals, and hydrolytic enzymes [21].

## 5. Conclusion

High-flux dialysis was not superior to low-flux dialysis in improving erythropoietin responsiveness and did not improve erythropoietin responsiveness in Arabic end-stage kidney disease patients even after the maintenance of hemodialysis for ≥1 year. It is apparent that high-flux dialysis may be better than low-flux dialysis regarding nutritional status, parathyroid hormone level, and blood cholesterol level, and further studies are required to evaluate that. Finally, erythropoietin resistance was associated with lower weight, longer hemodialysis vintage, lower hemoglobin, and higher NLR.

## Figures and Tables

**Table 1:** Comparison between the two groups regarding demographic data.

Variable	Group 1	Group 2	Test	*p* value
Age (years), mean ± SD	55.4 ± 15.3	51 ± 12	1.67	0.097
Male sex, no (%)	28 (56%)	48 (80%)	7.356	0.007^*∗∗*^
Weight (kg), median (IQR)	55.5 (50.5–65)	67.5 (48–79)	−3.471	0.001^*∗∗*^
BMI (kg/m^2^), median (IQR)	22.5 (19.1–23.7)	23.9 (19.7–27)	−2.738	0.006^*∗∗*^
SBP, mean ± SD	153 ± 16.55	157 ± 17	−1.336	0.184
DBP, mean ± SD	82.2 ± 13	86 ± 14.7	−1.397	0.165
IDWG (kg), median (IQR)	2 (1–4)	2 (1–3.5)	−0.874	0.382
Dialysis vintage (m), median (IQR)	24 (14.4–75)	48 (36–84)	−1.657	0.097

^*∗*^Significant, ^*∗∗*^highly significant, SD: standard deviation, IQR: interquartile range, BMI: body mass index, SBP: systolic blood pressure, DBP: diastolic blood pressure, IDWG: interdialytic weight gain, and m: months.

**Table 2:** Comparison between the two groups regarding laboratory data.

Variable	Group 1	Group 2	Test	*p* value
ALT (IU/L), median (IQR)	9.2 (5.3–11.3)	9.85 (6.7–12.7)	−1.249	0.212
Serum albumin (g/dL), median (IQR)	4.14 (3.9–4.3)	4.18 (3.9–4.3)	−0.757	0.449
URR (%), median (IQR)	65.3 (58.3–68)	61.1 (54.9–73)	−1.237	0.216
Uric acid (mg/dL), median (IQR)	6.43 (6.1–7.3)	6.9 (6.3–7.76)	−1.693	0.09
Hemoglobin (g/dL), mean ± SD	11.2 ± 1.24	11 ± 1.2	1.165	0.247
WBC (×10^3^/mm^3^), median (IQR)	5.6 (5–6.47)	5.5 (4.45–6.8)	−0.696	0.486
Neutrophil (×10^3^/mm^3^), median (IQR)	3.2 (2.56–4)	3 (2.4–4.26)	−0.408	0.683
Lymphocyte (×10^3^/mm^3^), median (IQR)	1.7 (1.3–2.1)	1.56 (1.2–1.8)	−1.729	0.084
Platelets (×10^3^/mm^3^), mean ± SD	189.6 ± 56	186 ± 51	0.326	0.745
NLR (%), median (IQR)	2 (1.2–2.7)	2.2 (1.67–2.63)	−1.213	0.225
PLR (%), median (IQR)	110 (86.6–129)	111 (94–158)	−1.297	0.195
Cholesterol (mg/dL), mean ± SD	169.7 (143–185)	154 (129–165)	−2.63	0.009^*∗∗*^
Triglycerides (mg/dL), median (IQR)	142 (82–245)	145 (89.4–192)	192.05	0.64
LDL (mg/dL), mean ± SD	90.48 (74–110)	82 (72–102)	−0.805	0.421
HDL (mg/dL), median (IQR)	35.6 (30–45)	36.5 (30–42)	−0.408	0.683
Calcium (mg/dL), median (IQR)	8.9 (8.6–9.2)	8.8 (8.5–9)	−1.755	0.079
Phosphorus (mg/dL), median (IQR)	4.9 (4–7)	4.99 (4–5.95)	−0.396	0.692
iPTH (pg/dL), median (IQR)	476 (268–1022)	296 (167–639)	2.774	0.006^*∗∗*^
Iron (*μ*g/dL), mean ± SD	53.6 (39.4–76)	51 (37.9–61)	−0.877	0.381
TSAT (%), median (IQR)	25 (18.85–44)	25 (18–29.8)	−1.237	0.216
Ferritin (ng/mL), median (IQR)	1165 (373–1883)	768 (544–1506)	−0.865	0.387
Folate (*μ*g/L), median (IQR)	13.6 (6.98–19.8)	10.1 (7.4–19.8)	−0.243	0.808
Vitamin B12 (pg/mL), median (IQR)	444.7 (369–704)	513 (391–775)	−0.744	0.457

^*∗*^Significant, ^*∗∗*^highly significant, IQR: interquartile range, SD: standard deviation, ALT: alanine transaminase, URR: urea reduction ratio, WBC: white blood cells, NLR: neutrophil-to-lymphocyte ratio, PLR: platelet-to-lymphocyte ratio, LDL: low-density lipoprotein, HDL: high-density lipoprotein, iPTH: intact parathyroid hormone, and TSAT: transferrin saturation.

**Table 3:** Univariate logistic regression analysis of the erythropoietin resistance index among all studied patients.

Parameter	*β*	OR	95% CI		*p*
			Lower	Upper	
Age (years)	−0.01	0.99	0.949	1.034	0.66
Weight (kg)	−0.022	0.979	0.954	1.004	0.1
BMI (kg/m^2^)	−0.037	0.964	0.88	1.055	0.425
Hemodialysis vintage (months)	0.117	1.124	0.979	1.291	0.097
IDWG (kg)	−2.074	0.126	0.043	0.371	≤0.001^*∗∗*^
ALT (U/L)	−0.053	0.949	0.856	1.052	0.316
Serum albumin (g/dL)	−3.397	0.033	0.003	0.348	0.004^*∗∗*^
eGFR (ml/min/1.73 m^2^)	0.019	1.019	0.704	1.475	0.92
Urea reduction ratio (%)	0.026	1.027	0.98	1.075	0.262
Serum uric acid (mg/dL)	0.341	1.407	0.884	2.24	0.15
Hemoglobin (g/dL)	−0.42	0.657	0.473	0.914	0.013^*∗*^
White blood cells (×10^3^/mm^3^)	0.234	1.263	0.884	1.805	0.199
Neutrophil (×10^3^/mm^3^)	0.429	1.535	0.934	2.524	0.091
Lymphocyte (×10^3^/mm^3^)	−0.02	0.98	0.329	2.917	0.971
Platelets (×10^3^/mm^3^)	0.002	1.002	0.992	1.012	0.742
NLR (%)	0.709	2.031	1.189	3.47	0.01^*∗*^
PLR (%)	0.005	1.005	0.998	1.013	0.184
Serum cholesterol (mg/dL)	0.015	1.015	0.998	1.032	0.086
Serum triglycerides (mg/dL)	−0.001	0.999	0.991	1.007	0.814
Low-density lipoprotein (mg/dL)	0.015	1.015	0.997	1.033	0.096
High-density lipoprotein (mg/dL)	0.03	1.03	0.969	1.095	0.339
Serum calcium (mg/dL)	0.005	1.005	0.516	1.958	0.989
Serum phosphorus (mg/dL)	−0.237	0.789	0.533	1.168	0.237
iPTH (pg/dL)	0.000	1	1.000	1.001	0.641
Serum iron (*μ*g/dL)	−0.048	0.953	0.919	0.988	0.01^*∗*^
Transferrin saturation (%)	−0.026	0.974	0.928	1.023	0.291
Ferritin (ng/mL)	0.001	1.001	1	1.002	0.021^*∗*^
Serum folate (*μ*g/L)	0.032	1.033	0.942	1.132	0.494
Serum vitamin B12 (pg/mL)	0.001	1.001	0.999	1.002	0.409

*β*: regression coefficient, OR: odds ratio, CI: confidence interval, BMI: body mass index, IDWG: interdialytic weight gain, ALT: alanine transaminase, eGFR: estimated glomerular filtration rate, NLR: neutrophil-to-lymphocyte ratio, PLR: platelet-to-lymphocyte ratio, and iPTH: intact parathyroid hormone.

**Table 4:** Multivariate logistic regression analysis of the erythropoietin resistance index among all studied patients.

Parameter	*β*	OR	95% CI		*p*
			Lower	Upper	
Weight (kg)	−0.035	0.966	0.94	0.992	0.011^*∗*^
Hemodialysis vintage (months)	0.158	1.172	1.036	1.325	0.012^*∗*^
Hemoglobin (g/dL)	−0.634	0.531	0.362	0.779	0.001^*∗∗*^
NLR (%)	0.89	2.436	1.321	4.493	0.004^*∗∗*^

*β*: regression coefficient, OR: odds ratio, CI: confidence interval, NLR: neutrophil-to-lymphocyte ratio, ^*∗*^significant, and ^*∗∗*^highly significant.

## Data Availability

The data supporting the findings of this study are available from the corresponding author upon reasonable request (waabdelhamid@medicine.zu.edu.eg).
